# Role of Two Types of Syntactic Embedding in Belief Attribution in Adults with or without Asperger Syndrome

**DOI:** 10.3389/fpsyg.2017.00743

**Published:** 2017-05-12

**Authors:** Morgane Clémentine Burnel, Marcela Perrone-Bertolotti, Stephanie Durrleman, Anne C. Reboul, Monica Baciu

**Affiliations:** ^1^Université Grenoble Alpes, CNRS, LPNC UMR 5105Grenoble, France; ^2^Université de Lyon, CNRS, Institute for Cognitive Sciences – Marc Jeannerod (UMR 5304)Bron, France; ^3^Department of Psycholinguistics, Faculty of Psychology and Educational Sciences, University of GenevaGeneva, Switzerland

**Keywords:** Theory of Mind, syntax, emergence, reasoning, metarepresentation, misrepresentation, autism spectrum disorders

## Abstract

The role of syntax in belief attribution (BA) is not completely understood in healthy adults and understudied in adults with autism spectrum disorder. Embedded syntax could be useful either for the development of Theory of Mind (ToM) (*Emergence* account) or more generally over the lifespan (*Reasoning* account). Two hypotheses have been explored, one suggesting that embedding itself (Relatives and Complement sentences and *Metarepresentation* account) is important for ToM and another one considering that the embedding of a false proposition into a true one (Complement sentences and *Misrepresentation* account) is important. The goals of this study were to evaluate (1) the role of syntax in ToM (*Emergence* vs. *Reasoning* account), (2) the type of syntax implied in ToM (*Metarepresentation* vs. *Misrepresentation* account), and (3) the verbally mediated strategies which compensate for ToM deficits in adults with Asperger Syndrome (AS). Fifty NeuroTypical (NT) adults and 22 adults with AS were involved in a forced-choice task including ±ToM tasks (BA and a control task, physical causation, PC) under four Interference conditions (silence, syllable repetition, relative sentences repetition, and complement sentences repetition). The non-significant ±ToM × Interference interaction effect in the NT group did not support the *Reasoning* account and thus suggests that syntax is useful only for ToM development (i.e., *Emergence* account). Results also indicated that repeating complement clauses put NT participants in a dual task whereas repeating relative clauses did not, suggesting that repeating relatives is easier for NT than repeating complements. This could be an argument in favor of the *Misrepresentation* account. However, this result should be interpreted with caution because our results did not support the *Reasoning* account. Moreover, AS participants (but not NT participants) were more disrupted by ±ToM tasks when asked to repeat complement sentences compared to relative clause sentences. This result is in favor of the *Misrepresentation* account and indirectly suggests verbally mediated strategies for ToM in AS. To summarize, our results are in favor of the *Emergence* account in NT and of *Reasoning* and *Misrepresentation* accounts in adults with AS. Overall, this suggests that adults with AS use complement syntax to compensate for ToM deficits.

## Introduction

The concept of Theory of Mind (ToM) was introduced by two primatologists, Premack and Woodruff (1978), and refers to the ability to attribute mental states (i.e., beliefs, thoughts, feelings, desires, emotions, or intentions) to others, in order to predict or explain their behavior. Using ToM, people are able to predict the output of a system (i.e., someone else’s behavior) from invisible states (i.e., mental states). Dennett (1978) explained that the only robust way of testing the attribution of mental states is to require people to predict a behavior from a belief or a representation about the world which may contrast with reality, that is to say a False Belief (FB). When evaluating True Beliefs (TB), because the belief is identical to reality, researchers cannot distinguish whether it is the reality state or the belief attribution (BA) (i.e., ToM) that is responsible for success.

A well-known result in the literature is the inability of children under 4 or 5 years to succeed at FB tasks (Yirmiya et al., 1998), indicating that before this age their ToM is not mature enough to allow them to attribute FBs to people and to use these attributions to predict behavior. The ability to pass FB tasks is considered as a milestone for ToM and ToM is mostly studied by means of FB tasks. This task nevertheless entails some limitations given that ToM is not limited to FB and FB is not limited to ToM (Bloom and German, 2000). Although (explicit) FB tasks are successfully performed around the age of 4, (implicit) FB tasks, where children are not specifically required to give an answer, can be performed before 2 years of age (see Onishi and Baillargeon, 2005; Southgate et al., 2007) but it is still debated if implicit and explicit tasks tap on the same processes (San Juan and Astington, 2012). Additionally, different studies showed that other (explicit) ToM tasks can be performed before the age of 4, for instance tasks evaluating the understanding of goals, desires, intentions, perceptions, feelings, knowledge, and ignorance (Wellman and Liu, 2004; Hutto et al., 2011). Additionally, ToM keeps on developing after childhood (Apperly, 2012; Brizio et al., 2015). Adolescents and adults perform correctly not only at FB tasks but also at other tasks evaluating the understanding of more complex social situations (Lawson et al., 2004; Beaumont and Sofronoff, 2008; Bosco et al., 2014). Thus, ToM is not a monolithic ability and the FB paradigm, although useful, does not cover all ToM processes. The second limitation underlined by Bloom and German (2000) is that FB tasks do not only measure ToM because they also involve other cognitive abilities such as attention, working memory and language. In order to succeed, children must pay attention to and subsequently recall the sequence of events, as well as understand the narrations and questions. It is worth noting that this second limitation is not applicable only to FB tasks but also to most of the tools designed to assess ToM as they often include long narrations (e.g., Social stories and Faux Pas test) or enhanced executive demands (e.g., Second order FB tasks). Nevertheless, studies have shown that verbal and non-verbal FB tasks are strongly correlated (Call and Tomasello, 1999). In the current study the choice to study BA (using a non-verbal paradigm) is mainly motivated by the difficulty to create appropriate tasks to assess ToM in adults without language as a confound. Although we are specifically interested in BA in this study, we are well aware that ToM is not limited to this ability, and that conclusions drawn on BA will have to be evaluated regarding other ToM abilities before being generalized to ToM.

Our study builds on the observation that children with Autism Spectrum Disorders (ASD) have a delayed ToM, as they succeed at FB tasks later than their NeuroTypical (NT) peers matched for intellectual abilities (see the meta-analysis of Yirmiya et al., 1998) and as they also succeed later than their NT peers on other ToM tasks. Happé (1995) showed that children with ASD need a higher mean verbal mental age than NT peers to succeed at FB tasks, namely that of 8 years and a half. Given this supplementary level of language required to perform FB tasks, Happé (1995) suggested that children with ASD use language to compensate their ToM deficits.

Asperger Syndrome (AS) is a particular form of ASD without intellectual or language delay. The formal diagnosis of AS which existed in the DSM-IV-TR (American Psychiatric Association, 2000) is no longer available in the DSM-5 (American Psychiatric Association, 2013), however, we maintain it in the current work because all of the participants included in this study received a diagnosis of “AS.” Children with a diagnosis of AS have better language abilities compared to children with other forms of autism (Manjiviona and Prior, 1999) they also show better social skills (Walker et al., 2004) and better performance at ToM tasks (Bowler, 1992). One goal of the current study is to test the hypothesis of verbally mediated strategies to compensate for ToM deficits in AS (Goal 3).

Different components of language could be useful for ToM in children, such as semantics, vocabulary, and syntax. The meta-analysis performed by Milligan et al. (2007) on 104 studies of NT children suggested that even if all of these language abilities are linked to FB understanding, some of them would be more useful than others. Indeed, the authors found that syntax accounted for 29% variance in FB scores whereas semantics and vocabulary accounted for 23 and 12% of variance, respectively. Thus, syntax is particularly important for ToM in NT children and could be an important element to promote ToM in ASD (Durrleman et al., 2016). Which syntactic structures are most important to support ToM in NT is still a question under investigation.

de Villiers and de Villiers (2000) proposed that a specific type of syntax used in Complement Sentences (CS) is particularly useful for FB. Indeed, sentential complements are often inserted in sentences with mental state verbs and serve to reflect the perspective of the subject of the matrix. For example, in: “*Sally thinks that the marble is in the basket*,” the underlined complement clause represents the subjective belief of Sally. Related to this is the fact that the embedded proposition can be true or false, independently of the entire sentence (just as beliefs may accurately reflect reality, or not). For example, in our previous example the complete sentence can be true (i.e., Sally really thinks that the marble is in the basket) and at the same time the embedded proposition can be false (i.e., the marble is not in the basket). In order to determine if the sentence is true or false, one should consider the mental world and not the real world. As proposed by de Villiers and de Villiers (2000), CS would therefore be an excellent tool for enabling children to represent FB.

Smith et al. (2003) proposed that another type of structure, mainly Relative Clause Sentences (RS) could also be important for FB reasoning. According to these authors, “Metarepresentation arises when a representation of an event is embedded inside a representation of an event,” thus embedding is what allows metarepresentation and any structure involving the embedding of events (thus metarepresentation) should be related to ToM. Consider the following example, with an underlined RS: “*Sally looked for the marble that Anne placed inside the basket.*” Just as in a CS, RS also includes an embedded proposition. However, in the case of CS, recall that the embedded proposition can be false with the entire sentence being true, while in a RS if the embedded proposition is false (i.e., the marble is not in the basket) then the entire sentence is also false (i.e., Sally cannot look for the marble that Anne placed in the basket if Anne didn’t place it in the basket).

*Misrepresentation* and *Metarepresentation* accounts are in opposition regarding syntactic structures that are important for ToM. According to the *Misrepresentation* account, what it important for ToM is the embedding of a false proposition inside a true one. Thus, stronger links should be found between ToM and CS than between ToM and RS. According to the *Metarepresentation* account, it is only the embedding that is important for ToM. Thus, the same links should be found between ToM and CS as between ToM and RS. Smith et al. (2003) showed in NT children that the understanding of certain RS was significantly linked to FB success. However, given that the study included no CS understanding task, results cannot guarantee that CS would not have been more closely related to ToM than RS. One goal of the current study is to compare the role of CS and RS in NT and in AS so as to evaluate *Misrepresentation* and *Metarepresentation* accounts (Goal 2). The comparison between the two populations will allow us to evaluate the existence of verbally mediated strategies for ToM in AS (Goal 3).

While the *Misrepresentation* and *Metarepresentation* accounts refer to the type of syntactic structure that is important for ToM, other hypotheses can be made regarding how these structures relate to ToM. Thus, apart from evaluating which element of language is the most important to support ToM, different hypotheses are made about the relationship between language and ToM. In particular, three accounts can be proposed. The first one is the *Reasoning* account, in which language would allow ToM reasoning over the entire lifespan. The second one is the *Emergence* account in which language is useful only for ToM development but not in adulthood (see Moses, 2001 for a similar account about the links between ToM and executive function).

According to a third account, the *Expression* account, the correlations found between language and ToM could arise from the verbal nature of the ToM tasks classically used (Miller, 2001; see also Moses, 2001). Indeed, language is first and foremost necessary to understand the narrations and instructions of these ToM tasks and as such directly affects ToM performance. We note that the *Emergence* and *Reasoning* accounts, as well as the *Expression* account can co-exist and are thus not mutually exclusive. Indeed, language could be a prerequisite for ToM (as suggested by *Reasoning* or *Emergence* accounts) and at the same time language could limit ToM performance due to the verbal instructions involved in ToM tasks (*Expression* account). However, when correlations are found between language and verbal ToM tasks we cannot eliminate the possibility that this link is only due to verbal instructions (i.e., *Expression* account). Consequently, to correctly evaluate the relationship between language and ToM (*Emergence* vs. *Reasoning* accounts), non-verbal ToM tasks are mandatory. The main goal of the current study is to evaluate *Reasoning* and *Emergence* accounts by mean of non-verbal tasks (Goal 1).

One way to disentangle the *Reasoning* and *Emergence* accounts is to study links between ToM and language in adults, because it is in adults that predictions differ. Indeed, in adults, according to the *Reasoning* account, language and ToM continue to be closely related. According to the *Emergence* account, however, no relation should be observed between ToM and language in adulthood. Studying the links between ToM and language in adults entails many challenges. First of all, classical FB tasks used in children cannot be used because they are too simple and adults would be at ceiling. Other tasks are available to assess ToM in adults (e.g., Social stories and Faux Pas test) but they generally include long narrations which are problematic regarding the *Expression* account. Similar limitations (facility of task) occur for the evaluation of *Misrepresentation* and *Metarepresentation* accounts in adults. In order to overcome these limits, two solutions have been proposed, one consisting of the study of brain-injured adults, and another one proposing the use of dual task paradigms, which is the method we adopt in the current study.

The majority of studies evaluating the relation between ToM and language at an adult age are performed in cognitively impaired patients after brain lesions, typically stroke patients. Indeed, the evaluation of patients with post-stroke aphasia and language deficits might be an important source of information on ToM functioning. Investigations have shown that, despite important syntactic deficits, post-stroke patients are able to perform ToM tasks (Varley and Siegal, 2000; Varley et al., 2001; Apperly et al., 2006). But as underlined by Caplan (1992), it is not clear what exactly is affected in such cases: linguistic performance or linguistic competence. If the patients tested in these experiments are affected in their linguistic performance through disrupted access, their linguistic competence might nevertheless be intact, allowing them to perform normally in ToM tasks.

Newton and de Villiers (2007) proposed a dual task paradigm in order to study the relation between language and ToM reasoning in healthy adults. The dual task consisted of the comparison of a non-verbal FB and a non-verbal TB task, during a verbal shadowing task and a non-verbal rhythmic task. The authors reported decreased performance for the FB condition but not for the TB condition, specifically during verbal shadowing but not during the non-verbal interference task. The authors concluded in favor of the role of language in BA. Forgeot d’Arc and Ramus (2011) highlighted different limitations to Newton and de Villiers’s conclusion, specifically criticizing the opposition between FB and TB. Historically, FB is the preferred indication of ToM over TB because, although a correct response to TB task can be achieved by means of ToM, it can also be achieved without ToM and thus a correct answer at a TB task does not always reflect ToM abilities. However, TB can still be achieved by means of ToM, and thus could reflect ToM processes too. Newton and de Villiers (2007) did not explain the reasons why language should be more useful during FB than during TB, and Forgeot d’Arc and Ramus (2011) considered that because both FB and TB are ToM they should be used jointly to assess ToM abilities. Moreover, according to Forgeot d’Arc and Ramus (2011), to prove a specific role of language during ToM, the interference effect of language on ToM should be greater than the interference effect of language on a matched task which does not require ToM. Thus, Forgeot d’Arc and Ramus (2011) proposed a different paradigm to assess the role of inner speech in attributing beliefs in adults. Similarly to Newton and de Villiers (2007), they used verbal shadowing as a dual task to inhibit inner speech. However, rather than contrasting TB and FB as in Newton and de Villiers (2007), they contrasted the ability to attribute (true or false) beliefs with the ability to attribute goals or to infer physical causation (PC). TB conditions were not used in isolation or contrasted to FB conditions, but as a means to control for response biases in FB conditions (see Forgeot d’Arc and Ramus, 2011, p. 977). Results reported for 58 NT adults showed that the role of inner speech in BA was not significantly different from the role of inner speech in goal attribution or in PC inference. Thus, they concluded that BA is not specifically dependent on inner speech. Overall, studies on NT adults are rather in favor of an *Emergence* account than a *Reasoning* account, suggesting that inner speech is not clearly implied in BA after childhood. No data is currently available in NT adults regarding the specific role of syntax (rather than inner speech) for ToM reasoning. More specifically, the *Misrepresentation* and *Metarepresentation* accounts have not yet been explored in a population of NT adults. Furthermore, these *Misrepresentation* and *Metarepresentation* accounts, as well as the *Emergence* and *Reasoning* accounts have yet to be examined in adults with ASD.

In the current study we had three main goals. Goal 1 was to assess *Emergence* and *Reasoning* accounts in NT adults, by mean of a dual task paradigm, to evaluate the relation between syntax and ToM. According to the *Emergence* account, language is not useful for ToM reasoning in adulthood. Thus, a verbal interference task should not disrupt the ability to attribute beliefs more than it disrupts the ability to perform a control task. In contrast, according to the *Reasoning* account, language is useful for ToM reasoning over the lifespan. In this case, a verbal interference task disrupts the ability to attribute beliefs, significantly more than it disrupts the ability to perform a control task. Goal 2 was to evaluate the *Metarepresentation* and *Misrepresentation* accounts in adults. According to the *Metarepresentation* account, the ability to embed a proposition into another is sufficient for ToM reasoning. Thus, being engaged in an interference task that involves RS should disrupt ToM as much as being engaged in an interference task that involves CS. In contrast, according to the *Misrepresentation* account, the most important linguistic structures for ToM are those embedding a false proposition in a true one. Thus, a dual task involving RS should not disrupt ToM as much as one involving CS. Goal 3 was mostly transversal and consisted in the evaluation of the hypothesis of a verbally mediated strategy to attribute beliefs in adults with AS. If adults with AS use language as a means to compensate for persistent ToM deficits, their ability to attribute beliefs when they are concurrently engaged in a verbal task should be significantly more disrupted than in NT adults. Put differently, we hypothesized that results in NT will be in favor of the *Emergence* account whereas results in AS will be in favor of the *Reasoning* account. The methodology of the current paper is a combination of paradigms used by Newton and de Villiers (2007) and Forgeot d’Arc and Ramus (2011). The difference from Newton and de Villiers (2007) and Forgeot d’Arc and Ramus (2011) is the evaluation of the role of specific syntactic structures rather than inner speech during BA. Moreover, we compared ToM and non-ToM tasks (as in Forgeot d’Arc and Ramus, 2011), as well as verbal and non-verbal interference tasks (as in Newton and de Villiers, 2007) in NT adults and in adults with AS.

## Materials and Methods

### Participants

Fifty-three NT adults and 25 adults with AS, all French native speakers, were initially included in the study. Three NT participants and three adults with AS were unable to perform all tasks so their data were excluded. We finally retained 50 NT participants (26 males, 24 females; mean age 21 years, *SD* 4.9) and 22 participants with AS (12 males, 10 females; mean age 32 years, *SD* 8.9). Participants provided written informed consent and the study was approved by the local ethical committee (CERNI, N° 2015-09-15-74). NT participants were students of the local university, while participants with AS were mainly recruited from the local Expert Center for AS diagnosis in adults. All of them completed the Hospital Anxiety and Depression scale (HAD) (Zigmond and Snaith, 1983) to assess possible anxiety and depression symptoms. This test was applied because AS are more prone to anxiety and depression (Stewart et al., 2006).

Recall that the main objective was to evaluate the interaction between ToM and syntactic abilities in NT and AS. ToM evaluation was based on the comparison of two experimental conditions, named ±ToM conditions. The +ToM condition was named BA and allowed ToM assessment, whereas the -ToM condition was named PC and hence was the control condition. ±ToM conditions were performed under four interference conditions, three verbal tasks [involving a series of syllables (SS), RS or complement clause sentences] and Silence.

### ToM Evaluation: Stimuli and Tasks

**FIGURE 1:**
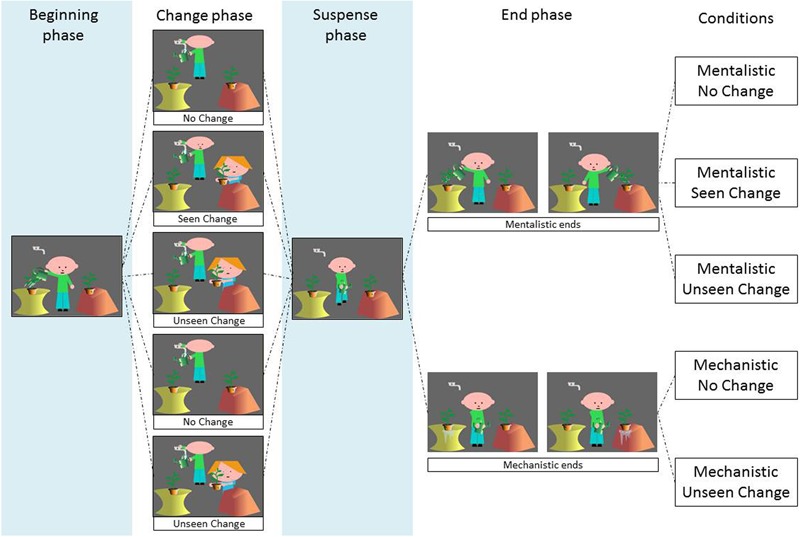
**Examples of cartoons presented during the four phases (beginning, change, suspense and possible ends) according to five experimental conditions (adapted from Forgeot d’Arc and Ramus, 2011).** The combination of three change phases (No Change, Seen Change, and Unseen Change) with two different end phases (Mentalistic ends and Mechanistic ends) allows the creation of five conditions (Mentalistic No Change, Mentalistic Seen Change, Mentalistic Unseen Change, Mechanistic No Change, and Mechanistic Change). During each end phase, two pictures are presented at the same time and the participant is asked to choose the correct ending.

Stimuli used during BA and PC were cartoons similar to those presented in Forgeot d’Arc and Ramus (2011). Seventy-five cartoons representing 15 scenarios were presented to participants during the ±ToM conditions. Each cartoon was composed of four successive phases: *beginning, change, suspense* and *pair of possible ends* (one correct and another incorrect, see description below and **Figure 1**). Participants were instructed to choose the correct ending from the two presented (i.e., a forced-choice task). In the *change* phase, cartoons were presented in three situations (No Change, Change Seen, and Change Unseen). According to the *pair of possible ends* cartoons were presented in two situations (Mentalistic end and Mechanistic end), leading to a total of five situations: *Mentalistic No Change, Mentalistic Seen Change, Mentalistic Unseen Change, Mechanistic No Change*, and *Mechanistic Unseen Change* (see **Figure 1** for details and Forgeot d’Arc and Ramus, 2011, pp. 978–980).

The *beginning* phase was identical to all five situations and represented the general context and the main agent of the scenario, i.e., “man standing between two plants, holds a watering can; there is a faucet in the background; the man waters the plant on the left and then leaves the scene to fill his watering can”.

As mentioned in **Figure 1**, the *change* phase is presented in three versions. In the *No Change* situation, nothing happens after the agent fills his watering can. In the *Change Seen* situation (i.e., TB) a change occurs and is perceived by the agent. Specifically, this change consists of a woman appearing in the scene, who switches the two plants while the man is watching her. In the *Unseen Change* situation (i.e., FB) the change is identical as in the previous situation, except that it is not seen by the agent (see **Figure 1**). The *suspense* phase is identical across all situations and consists in the agent’s action (e.g., after having filled the watering can, he is standing between the two plants). The *end* phase can be *Mentalistic* or *Mechanistic*, each one proposing two choices. For the *Mentalistic* type, the choice concerns the agent’s action (e.g., watering the plant) either on the left or on the right. For the *Mechanistic* type, the choice concerns the mechanical action (e.g., water leaking from the pot) either on the left or on the right (see **Figure 1**).

Cartoons represented 15 scenarios, each of them being declined in five experimental situations (i.e., *Mentalistic No Change, Mentalistic Seen Change, Mentalistic Unseen Change, Mechanistic No Change*, and *Mechanistic Unseen Change*) leading to a total of 75 cartoons. Cartoons were presented in five Cartoon blocks. Each Cartoon block contained 15 cartoons, including one occurrence of each scenario; each experimental situation was presented three times in each Cartoon block (of three different scenarios). The number of correct answers per participant and per condition was recorded. The number of correct answers in pairs of situations was then used, as in Forgeot d’Arc and Ramus (2011), to compute two sensitivity indices during data processing (signal detection analysis). The assessment of BA was based on answers in the *Mentalistic Seen Change* and *Mentalistic Unseen Change* conditions whereas the assessment of PC was based on answers in the *Mechanistic No Change* and *Mechanistic Unseen Change* conditions (see Data Scoring section).

### Interference Evaluation: Stimuli and Tasks

We created verbal material to assess interference processes between syntactic processes and ToM tasks described previously. Interferences were manipulated in four experimental conditions as mentioned previously (three verbal and one silent). For the verbal conditions we used Complement sentences (CS) and Relative Sentences (RS). We evaluated which type of sentence, CS or RS, is the most useful to ToM. A third verbal condition was represented by a Series of syllables (SS) as a control condition without syntax but requiring the phonological buffer. Finally, a Silence condition was proposed as a control.

A total of 252 stimuli (84 CS, 84 RS, and 84 SS) were created and presented during the three verbal conditions (see **Table 1**). The number of syllables (i.e., 11) was controlled across conditions. CS and RS were built as pairs (see **Table 1**), differing only in terms of syntax but remaining similar in terms of vocabulary, frequency of occurrence (LEXIQUE database, New et al., 2001) and plausibility (based on a preliminary experiment on a different group of participants, *t*(54) = -0.25, *p* = 0.80]. SS stimuli consisted of the same syllable (e.g., BA) repeated 11 times. Stimuli of CS, RS, and SS were incorporated in the Voxygen vocal synthesizer allowing the generation of three audio files, one for each verbal condition, having the same duration (7 min).

**Table 1:** Examples of complement sentences, relative sentences, and series of syllables, presented as triplets.

Complement sentence	Relative sentence	Series of syllables
Tu rapportes que des étoffes drapent la sculpture /ty 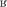 apɔ 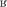 tə kə dez- etɔf d 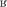 ap la skylty 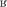 / You report that cloth drapes the sculpture	Tu répares les étoffes qui drapent la sculpture /ty 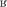 ep 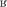 a lez- etɔf ki d 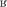 ap la skylty 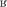 / You repair cloth that drapes the sculpture	/bi bibi bi bi bibi bi bi bibi/

The 252 stimuli were split into three Interference blocks: Complements block, Relatives block, and Syllables block. Interference blocks were presented concomitantly with the cartoons described above and presented for ToM evaluation. Using the same 252 stimuli, we also built three No-Interference blocks, each including 28 CS, 28 RC, and 28 SS. No-Interference blocks were used in isolation without cartoons, that is to say without any concomitant evaluation of ToM.

### Experimental Procedure

The procedure is described in **Table 2** and consists in three phases: Training, No-Interference, and Interference.

**Table 2:** Conduct of the experimental procedure.

	Training phase	No-Interference phase	Interference phase (random order)			
Material	1/3 No-Interference blocks	1/3 No-Interference blocks	–	Syllables block	Relatives block	Complements block
			1/5 Cartoon blocks	1/5 Cartoon blocks	1/5 Cartoon blocks	1/5 Cartoon blocks

*During the Training phase participants read sentences, during the No-Interference phase they repeated complement and relative sentences as well as a series of syllables and during the Interference phase participants completed Theory of Mind and language tasks concurrently.*

#### Training Phase

Each participant started with a short training session (5 min) consisting in reading aloud written CS and RS from one of the three No-Interference blocks (i.e., a total of 56 sentences to read per participants) to become familiar with the type of sentence and vocabulary.

#### No-Interference Phase

The No-interference experiment (7 min) started right after this short training. Participants were required to listen to recorded CS, RS, and SS within a No-Interference block (different from the one they read during the Training phase) and repeat what they heard after the end of each sentence (i.e., a total of 84 sentences or SS per block). Participants were recorded while repeating sentences or syllables. Their performance was evaluated in terms of error rates (%ER) in repetition.

#### Interference Phase

Each participant performed ToM tasks in four Cartoon blocks according to CS, RS, SS, and Silence conditions. The association between Cartoon block and Interference conditions, as well as the order of Interference conditions, was counterbalanced across participants. The order of cartoon presentation inside each block was randomized. During the Silence, participants were asked to choose as quickly as possible the end which best completed a presented cartoon. Responses were provided by means of two manual keys on a keyboard (“A” for the end presented on the left side of the screen and “P” for the end presented on the right side of the screen, on an AZERTY keyboard). Similarly, during the three linguistic conditions (i.e., Complements Repetition, Relatives Repetition, and Syllables Repetition), participants were asked to choose as quickly as possible the end which best completed a presented cartoon (pressing the same manual keys as presented above) with concomitant repetition of a heard sentence or SS (i.e., dual task paradigm). Participants were explicitly told that their answer would not be taken into account if they did not repeat what they heard. During these three verbal conditions, the recording of the audio file started with the first cartoon and continued until all 15 cartoons of the Cartoon block were completed. The Interference phase lasted approximately 30 min.

### Data Scoring and Analyses

#### Data Scoring

Correct answers and mean reaction times (RTs) were recorded for the five different cartoon situations (i.e., *Mentalistic No Change, Mentalistic Seen Change, Mentalistic Unseen Change, Mechanistic No Change*, and *Mechanistic Unseen Change*) with a maximum of three correct answers per situation. To simplify data analysis we computed two sensitivity indices (i.e., BA and PC) among the three initially proposed by Forgeot d’Arc and Ramus (2011). Thus, even if participants answered the *Mentalistic No Change* situations, these answers were not taken into account for analyses. To compute the BA sensitivity index we considered as hits correct answers at the *Mentalistic Unseen Change* situation, and as false alarms incorrect answers at the *Mentalistic Seen Change* situation. The PC index was computed from correct answers at the *Mechanistic Change* situation (i.e., Hits) and incorrect answers at the *Mechanistic No Change* situation (i.e., False alarms). There was a total of eight indices per participant: two per Interference condition (i.e., one for BA and one for PC) with a total of four Interference conditions (i.e., CS condition, RS condition, SS condition, and Silence condition).

Responses from No-Interference and Interference phases were recorded and the task execution was evaluated in terms of percentage of error in repetition. This was acted out by syllables repeated incorrectly or not repeated at all. A syllable was considered as incorrectly repeated if more than one phoneme was omitted or pronounced incorrectly. We computed the percentage of mispronounced syllables over the total number of syllables to repeat. Error rates for repetition were computed for CS and for RS.

We considered three Independent Variables, the Group (i.e., NT and AS), the ±ToM condition (BA and PC), and the Interference (i.e., Complements Repetition, Relatives Repetition, Syllables Repetition, and Silence) and three Dependent Variables [i.e., Signal detection indices (d’), RTs, and Error Rates during repetition (%ER)].

#### Data Analyses

By means of ANOVAs we begin with the evaluation of the possible difference between NT and AS groups in terms of age and in terms of anxiety and depression symptoms as assessed by the HAD scale. Significant differences were obtained thus we computed mean values of d’, RTs, and %ER for each participant and tested Spearman correlations with age, anxiety and depression scores in order to evaluate if being more prone to anxiety and depression would have an influence on participants’ performances.

We evaluated if each sensitivity indices (eight per participant according to two ±ToM conditions and four Interference conditions) was significantly above zero by mean of *t*-tests, in order to know if participants were above chance level. This was performed for NT and AS groups separately.

Then we performed an ANOVA on d’ and RTs in order to control experimental interference induction. We computed a three-way 2 × 2 × 4 ANOVA including Group (NT and AS) as between subject factor, two within-subject factors the ±ToM (BA and PC) and the Interference (Complements Repetition, Relatives Repetition, Syllables Repetition, and Silence) on signal detection indices as well as on RTs.

First, the effect of ±ToM was computed on d’ and RTs to compare our results with the previous results of Forgeot d’Arc and Ramus (2011) who found that performances on PC were better than performances on BA, and that participants took longer to answer on BA compared to PC. We also computed the Group ×±ToM interaction in order to determine if AS participants performed differently from NT participants on the BA task but not on the PC task. We then computed the Group × Interference ×±ToM interaction effect on d’ and RTs so as to evaluate our hypothesis of verbally mediated strategies compensating for ToM deficits in AS. In addition, to evaluate hypotheses that specifically concerned the NT group, we computed the Interference × ±ToM interaction effect on d’ and RTs within the NT group by means of planned comparisons. To assess the *Emergence* and *Reasoning* accounts, planned comparisons evaluated the difference between Syllables Repetition vs. Relatives Repetition and Syllables Repetition vs. Complements Repetition. To examine the *Metarepresentation* and *Misrepresentation* accounts, planned comparisons evaluated the difference between Relatives Repetition vs. Complements Repetition.

To finish, a three-way 2 × 2 × 2 ANOVA including Group (NT and AS) as between subject factor and two within-subject factors, the Interference phase (Interference and No-Interference) and the Syntax (CS and RS), was conducted on %ER.

We computed the Group effect in order to evaluate if AS participants were more prone to errors in repetition compared to NT participants. Furthermore, we computed the Syntax effect in order to evaluate if CS were more difficult to repeat than RS in line with the *Metarepresentation* account. We computed the Interference phase effect in order to evaluate if participants committed more errors in repetition during the Interference phase compared to the No-Interference phase. Finally, we evaluated the hypothesis of verbally mediated strategies in AS by the Group × Syntax and Group × Syntax × Interference phase interaction.

## Results

### Effect of Control Variables and Control of Experimental Interference Induction

Participants with AS were significantly older than NT [*F*(1,70) = 51.21, *p* < 0.05] and they reported significantly more anxiety and depression symptoms (Mean = 15, *SD* = 1.1 point) than NT participants (Mean = 9.5, *SD* = 0.7 point) [*F*(1,70) = 16.8, *p* < 0.05]. Spearman correlations indicated that there was no significant correlation between Age and the mean values of d’ [*r*(1,72) = 0.18, *p* = 0.12], RTs [*r*(1,72) = -0.17, *p* = 0.15], or %ER [*r*(1,61) = 0.10, *p* = 0.46] during sentence repetition. Similarly, there was no significant correlation between HAD scores and the mean values of d’ [*r*(1,72) = 0.12, *p* = 0.33] but there was a significant correlation between HAD scores and mean RTs [*r*(1,72) = 0.39, *p* < 0.05] with a tendency for more depressed and anxious participants to answer more slowly than less depressed and anxious participants. There was no significant correlation between HAD scores and the error rate during sentences repetition [*r*(1,61) = 0.07, *p* = 0.62].^1^

Sensitivity indices were computed for BA (i.e., Theory of Mind – ToM task) and PC (i.e., control task) in four Interference conditions (i.e., Complements Repetition, Relatives Repetition, Syllables Repetition, and Silence). *T*-tests results showed that the eight d’ were significantly greater than zero in the NT group (all *t*-values > 6.31, *p* < 0.05) as in the AS group (all *t*-values > 5.82, *p* < 0.05).

**FIGURE 2:**
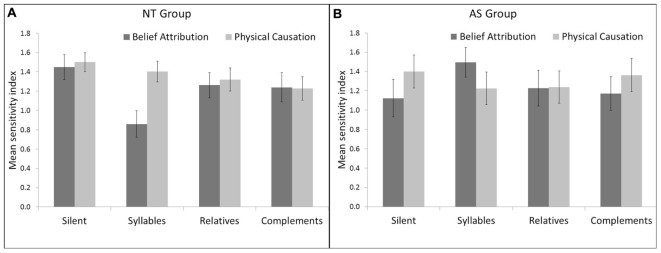
**Mean Indices and Standard Deviations (SD) in the NeuroTypical (NT) group (A)** and in the Asperger Syndrome (AS) group **(B)** according to Interference (Silent, Syllables, Relatives, and Complements) and ToM condition (Belief Attribution and Physical Causation).

In the NT group the effect of Interference on d’ was significant [*F*(3,147) = 2.07, *p* < 0.05]. The planned comparison between Complements Repetition (Mean = 1.25, *SD* = 0.09) vs. Silence (Mean = 1.41, *SD* = 0.07) was significant [*F*(1,49) = 5.28, *p* < 0.05] just as between Syllables Repetition (Mean = 1.20, *SD* = 0.07) vs. Silence [*F*(1,49) = 14.27, *p* < 0.05] whereas the planned comparison between Relatives Repetition (Mean = 1.28, *SD* = 0.07) vs. Silence was not significant [*F*(1,49) = 2.72, *p* = 0.11] (see **Figure 2**). Moreover in the NT group the effect of Interference on RTs was non-significant [*F*(3,147) = 0.71, *p* = 0.55] as for planned comparisons between the silent and verbal conditions [All *F*(1,49) < 1].

In the AS group, the effect of Interference was not significant either on d’ and RTs [All *F*(3,63) < 1] as for the planned comparisons between the silent and verbal conditions [All *F*(1,49) < 1].

### Results Provided by Signal Detection and Reaction Times Analyses

Despite a tendency for participants to obtain higher sensitivity indices on the PC condition (Mean = 1.35, *SD* = 0.06) compared to the BA condition (Mean = 1.22, *SD* = 0.06) the effect of ±ToM was not statistically significant [*F*(1,70) = 1.91, *p* = 0.17]. Nevertheless, participants answered significantly faster [*F*(1,70) = 114.8, *p* < 0.05] in the BA condition (Mean = 2.64 s, *SD* = 0.12) compared to the PC condition (Mean = 3.25 s, *SD* = 0.15).

**FIGURE 3:**
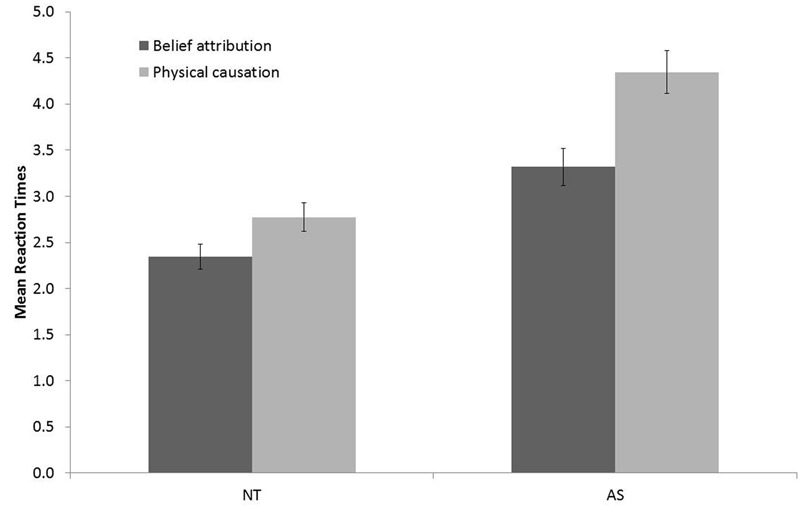
**Mean Reaction times, RTs (in seconds) depending on Group (NT and AS) and ±ToM condition (Belief Attribution and Physical Causation)**.

The Group ×±ToM interaction was not significant on d’ [*F*(1,70) < 1] suggesting that AS and NT participants succeeded equally to the ±ToM tasks. The Group × ±ToM interaction on RTs was significant indicating a difference of response speed between ±ToM conditions greater in AS than in NT (see **Figure 3**).

In order to evaluate our hypothesis of verbally mediated strategies to compensate for ToM deficits in AS we computed the Group × Interference ×±ToM interaction effect on d’ then on RTs.

The interaction for Group × Interference ×±ToM was significant on d’ [*F*(3,210) = 2.92, *p* < 0.05] suggesting that regarding signal detection indices the Interference ×±ToM interaction effect was different for NT and AS. Planned comparisons on d’ showed significant differences between Silence vs. Syllables Repetition [*F*(1,70) = 7.80, *p* < 0.05] and Syllables Repetition vs. Complements Repetition [*F*(1,70) = 6.27, *p* < 0.05] (see **Figure 2**). The difference between Syllables Repetition vs. Relatives Repetition was non-significant [*F*(1,70) = 3.42, *p* = 0.07] as was also the case for Silence vs. Relatives Repetition [*F*(1,70) < 1], Silence vs. Complements Repetition [*F*(1,70) < 1], and Relatives Repetition vs. Complements Repetition [*F*(1,70) < 1].

The interaction for Group × Interference ×±ToM was non-significant on RTs [*F*(3,210) = 1.47, *p* = 0.22]. A non-significant difference was obtained for each planned comparison performed.

Within the NT group, the Interference ×±ToM interaction effect on d’ was non-significant [*F*(1,70) = 2.55, *p* = 0.06]. Planned comparisons on d’ indicated significant differences between Syllables Repetition vs. Relatives Repetition [*F*(1,70) = 4.52, *p* < 0.05], Syllables Repetition vs. Complements Repetition [*F*(1,70) = 6.12, *p* < 0.05], and a non-significant difference between Relatives Repetition vs. Complements Repetition [*F*(1,70) < 1] (see **Figure 2**).

Within the NT group, the Interference ×±ToM interaction effect on RTs was non-significant [*F*(1,70) = 1.05, *p* = 0.37] as for the planned contrasts between Syllables Repetition vs. Relatives Repetition [*F*(1,70) < 1], Syllables Repetition vs. Complements Repetition [*F*(1,70) = 1.38, *p* = 0.24], and Relatives Repetition vs. Complements Repetition [*F*(1,70) < 1].

Because the significant Group × Interference ×±ToM interaction effect indicated that Interference ×±ToM interaction effect was different between groups, we also computed this effect within the AS group by mean of contrasts. The Interference × ±ToM interaction effect within the AS group was non-significant on d’ [*F*(1,70) = 1.18, *p* = 0.35] as on RTs [*F*(1,70) < 1].

### Results Provided by the %ER Analysis

**FIGURE 4:**
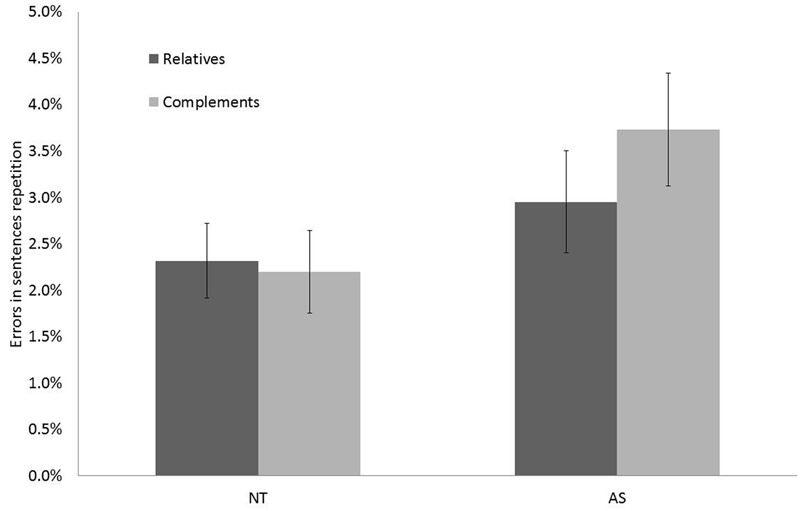
**Percentage of errors (%ER) in Sentence Repetition according to Group (NT, and AS) and Syntax (Relatives and Complements)**.

**FIGURE 5:**
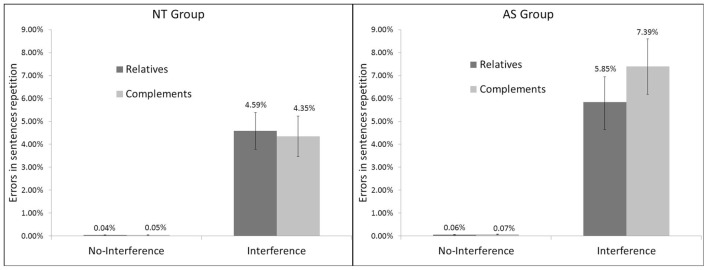
**Percentage of errors (%ER) in Sentence Repetition according to Group (NT and AS), Syntax (Relatives and Complements), and Interference phase (No-Interference and Interference)**.

ANOVAs on %ER during repetition showed a non-significant main effect of Group [*F*(1,61) = 2.45, *p* = 0.12], a non-significant main effect of Syntax (i.e., Syntactic structures) [*F*(1,61) = 6.30, *p* = 0.09] and a significant effect of Interference phase [*F*(1,61) = 2.45, *p* < 0.05] with participants committing more errors in repetition during the Interference phase (mean = 5.55%, *SD* = 0.69) compared to the No-Interference phase (mean = 0.05%, *SD* = 0.01). There was a significant Group × Syntax interaction on %ER [*F*(1,61) = 5.32, *p* < 0.05] indicating that the difference in error rates between the two Syntax conditions was greater in AS with more errors during CS repetition compared to RS repetition (see **Figure 4**). The Group × Syntax × Interference phase interaction effect was also significant [*F*(1,61) = 5.36, *p* < 0.05] and indicated that the %ER was greater in the Interference than in the No-Interference phase for the AS group compared to the NT group, with more errors during CR compared to RS repetition (see **Figure 5**).

In addition, the interaction HAD × Syntax on %ER during repetition was not significant [*F*(1,61) = 1.51, *p* = 0.22] and neither was the interaction HAD × Syntax × Interference [*F*(1,31) = 1.50, *p* = 0.23] suggesting that the Group × Syntax and Group × Syntax × Interference effects were not explained by the greater level of depression and anxiety in AS participants.

## Discussion

This study evaluated the relation between syntax and BA in NT and AS adults via a dual task paradigm. Participants performed ±ToM tasks involving BA (test condition) and PC (control condition) under four interference conditions, three verbal (Syllables Repetition, Relatives Repetition, Complements Repetition) and one Silent (i.e., control) Condition. Our goals were to assess (1) *Emergence* and *Reasoning* accounts in NT adults, (2) *Metarepresentation* and *Misrepresentation* accounts in NT adults, and (3) verbally mediated strategies to compensate ToM difficulties in adults with AS. Only major results will be discussed.

### *Emergence* vs. *Reasoning* Accounts in NT Adults

The first goal of this study was to evaluate the *Emergence* and *Reasoning* accounts that have different predictions regarding the usefulness of language for ToM reasoning during adulthood. We argued that if syntax is specifically useful for ToM reasoning, in NT adults a verbal interference task should disrupt the ability to attribute beliefs more than it disrupts the ability to perform a control task. Moreover, this interference should be more important for syntactic tasks (i.e., Complements Repetition or Relatives Repetition) than for a control interference task (i.e., Syllables Repetition). This second point is mostly discussed in the next section (see section *Metarepresentation* Vs *Misrepresentation* Accounts in NT Adults).

In the NT group, there was a significant effect of Interference on sensitivity indices but not on RTs. Moreover, results revealed no significant interaction between Interference and ToM both in terms of sensitivity indices and in terms of RTs. This suggests that even if being involved in a verbal interference task could disrupt the ability of individuals with NT to attribute beliefs, this disturbance is not specific to ToM (i.e., BA) as it was also observed for other tasks without a ToM dimension (i.e., PC). Thus, as mentioned in previous studies on NT adults (Newton and de Villiers, 2007; Forgeot d’Arc and Ramus, 2011), we found no specific need for language during ToM compared to control tasks. This result does not support the *Reasoning* account and thus suggests that according to the *Emergence* account, language, and more specifically syntax, is useful only for ToM development.

### *Metarepresentation* vs. *Misrepresentation* Accounts in NT Adults

Our second goal was to evaluate the *Metarepresentation* and *Misrepresentation* accounts. According to the *Metarepresentation* account, the ability to embed a proposition into another is sufficient for ToM reasoning. Thus, being engaged in an interference task that involves RS (i.e., Relatives Repetition) should disrupt ToM as much as being engaged in an interference task that involves CS (i.e., Complements Repetition), since both of these structures involve embedding. In contrast, according to the *Misrepresentation* account, the most important linguistic structures for ToM are those embedding a proposition with an independent truth-value. Thus, a dual task involving RS should not disrupt ToM as much as one involving CS. Moreover, both Relatives Repetition and Complements Repetition should disrupt ToM more than Syllables Repetition.

Results of signal detection analyses (**Figure 2**) showed that NT participants performed better in the ±ToM conditions (i.e., BA and PC) when they were silent compared to when they repeated SS or CS, but not when they repeated RS. Thus, the delayed repetition of a SS, or of sentences other than RS, put NT participants in a dual task situation. This result is important, given that in previous studies (Newton and de Villiers, 2007; Forgeot d’Arc and Ramus, 2011) participants performed a continuous shadowing task that could be more difficult than a delayed repetition task. So, repeating CS but not repeating RS, disrupted the ability of NT participants to choose the right end of cartoons during the experiment. Sentences in these two conditions were as close as possible, they included the same number of syllables, the same vocabulary and were judged as similarly plausible in a preliminary experiment. The fact that repeating CS put participants in a dual task situation whereas repeating RS did not, might indicate that repeating RS is easier for NT than repeating CS, and thus, an argument in favor of the *Misrepresentation* account. However, this result should be interpreted with caution given that (1) planned comparison for RS and CS on the sensitivity indices was not significant, and (2) there was no argument in favor of a specific role of language during BA compared to PC (see section *Emergence* Vs *Reasoning* Accounts in NT Adults).

### Verbally Mediated Strategies to Compensate ToM Difficulties in Adults with AS

We will first present results about differences between groups regarding general performances, and later discuss results regarding the hypothesis of verbally mediated strategies to compensate for ToM deficits in adults with AS.

In terms of signal detection analyses, no difference was obtained between AS and NT (see **Figure 2**). Thus, adults with AS did not show a ToM deficit on BA. This result is not surprising given that previous studies showed that people with ASD were able to perform FB tasks from 8 years of verbal mental age (Happé, 1995). It is important to note that even if participants with AS performed equally well to NT participants at this task, it does not mean that they have the same ToM abilities. Indeed, they could still be less performant on harder ToM tasks (e.g., second order FB, faux pas, etc.). Furthermore, participants with AS could have reached the same level of success as NT participants at these tasks using different strategies, that is to say offset strategies.

Adults with AS were also significantly slower than NT participants at answering. As illustrated in **Figure 3**, both NT and AS were significantly slower to perform PC than BA. This result is in line with Forgeot d’Arc and Ramus (2011) and could be explained by the fact that a majority (i.e., 3/5) of the videos involved Mentalistic ends with a requirement of attributing mental states to characters. Thus, participants who were trained to automatically attribute mental states, had to reevaluate the situation when they were confronted with mechanistic ends and this process might take supplementary information processing. Compared to NT, participants with AS showed increased RT differences between ToM conditions (i.e., BA and PC) (see **Figure 3**), possibly due to increased latency to refocus their attention on Mechanical indices rather than on Mentalistic ones. It could thus be argued that AS participants took more time to answer because ToM tasks were more difficult for them compared to NT adults even though the response accuracy of these groups was similar. The fact that participants with AS are slower than NT participants to answer (see **Figure 3**) is a commonly observed result (Bowler, 1997; Kaland et al., 2002). Given that HAD scores reflected that participants with AS were significantly more depressed and anxious than NT participants, and given that there was a significant correlation between HAD scores and RTs, this might also explain longer latency in AS than in NT (Emerson et al., 2005).

Our third goal was mostly transversal and consisted in the evaluation of the hypothesis of verbally mediated strategies to attribute beliefs in adults with AS. If adults with AS use syntax as a way to compensate for their ToM deficits, their ability to attribute beliefs when they are concurrently engaged in a verbal task should be significantly more disrupted than in NT adults.

The interaction for Group × Interference ×±ToM was significant regarding sensitivity indices (see **Figure 2**). This suggests that the dual task effect on sensitivity indices was different in NT and AS participants. According to the hypothesis of verbally mediated strategies to compensate ToM deficits in adults with AS, we hypothesized that the differences between the ±ToM conditions would be more important for Complements Repetition and for Relatives Repetition compared to Silence or compared to Syllables Repetition. The results did not support our hypothesis as we obtained an unexpected result for the repetition of a SS. Indeed, the SS repetition lead to a greater decline in performance for BA compared to PC in the NT group compared to the AS group (see **Figure 2**). Put differently, repeating syllables disrupted ToM (i.e., BA) in NT compared to a control task (i.e., PC) and to other dual tasks, but not in AS. We interpret this result as reflecting a higher memory load in Syllables Repetition than in the other conditions. Indeed, the Syllables Repetition condition was added as a rhythmic task involving the articulatory loop which stores and manipulates speech-based material. In a sentence made up of 11-syllables, syllables can be grouped together by words (e.g., 8 words in our material), and words can be grouped together according to syntax (i.e., six units in our material). Semantics and syntactic strategies create a reduced number of elements to remember for participants (Baddeley, 2000). However, when presented with a series of 11 syllables, participants could not create chunks to help them to remember the syllables. Participants were not informed that all series contained 11 syllables either. In light of this, repeating syllables arguably required significantly more memory load, both because they did not know how many syllables were to be repeated and could not apply semantic or syntactic strategies to create chunks of syllables.

Furthermore, another unexpected result is that in the AS group, the Interference effect and the interaction Interference ×±ToM were significant neither in terms of sensitivity indices (see **Figure 2**) nor of RTs. Thus, repeating SS, RS, or CS did not disrupt the ability to BA or to infer PC in participants with AS.

Participants with AS show deficits in executive functioning (Ozonoff et al., 1991, 2000; Hill, 2004) so compared to NT, their performance should be more disrupted during a dual task. In some studies, people with ASD were shown to be more sensitive to dual tasks (García-Villamisar and Sala, 2002; Lidstone et al., 2010). However, other studies have revealed that individuals with ASD (i.e., not specifically in AS) are not necessarily as affected as their NT peers during dual tasks. Previous studies using a dual task paradigm to evaluate the role of inner speech during executive functioning showed that children (Whitehouse et al., 2006) and adults (Wallace et al., 2009) with ASD were not disrupted as much as NT peers by articulatory suppression, arguing for limitations in the use of inner speech for executive functioning in ASD. Williams et al. (2008) showed intact inner speech use in ASD during a short-term memory task whereas García-Villamisar and Sala (2002) showed that adults with ASD were as disrupted as NT peers during executive tasks. Thus, previous results on the effect of verbal dual tasks in participants with ASD showed mixed results and it is currently difficult to have a clear overview of the field because populations (i.e., ASD with or without language delay, with or without intellectual delay) and hypotheses (i.e., role of inner speech during executive, working memory, or ToM tasks) varied amongst studies.

Recall that one of our goals was to evaluate the role of two syntactic structures during BA. We thus proposed a delayed repetition instead of a verbal shadowing task. Moreover, our participants were adults with AS, that is to say adults with autism without any language or intellectual delay. Lidstone et al. (2009) showed that inner speech impairment in children with autism is associated with greater non-verbal than verbal skills and AS is characterized by greater verbal than non-verbal skills (Chiang et al., 2014). Our result indicating that participants with AS were less sensitive to dual task effects could be explained by (1) a deficit in inner speech use during ToM or (2) by an expertise in inner speech use during ToM. Indeed, if participants with AS usually use language as a strategy to compensate their ToM deficits, they could be more used to solving ToM tasks while they are concurrently engaged in a verbal task, as compared to NT peers. Interestingly, if repeating RS or CS did not lead to a specific decrease in performance during ±ToM tasks, being involved in such tasks disrupted participants’ ability to repeat CS more than to repeat RS, during the Interference phase but not during the No-Interference phase. This result may be interpreted to suggest a specific role of CS during ±ToM tasks, along the lines of that predicted by the *Misrepresentation* account.

Based on the percentage of errors in repetition (%ER), results showed that the difference between CS and RS was greater in AS than in NT. Indeed, AS participants showed more errors during CS compared to RS repetition (see **Figure 4**), so adults with AS were more taxed than those with NT in CS repetition than in RS repetition, but crucially this was only during the Interference phase and not during the No-Interference phase. Thus, despite being able to repeat CS equivalently to RS as NT participants during the No-Interference phase, AS participants (but not NT participants) were more disrupted by ±ToM tasks (i.e., BA and PC) when asked to repeat CS compared to RS. This result is in favor of the *Misrepresentation* account and indirectly suggests verbally mediated strategies for ToM in AS (see **Figure 5**). Further studies are nevertheless needed in order to evaluate if this result is specific to ToM tasks (i.e., BA) compared to control tasks (i.e., PC). Moreover, because possible differences in IQ, executive function or language abilities between AS and NT were not examined in this study, it is thus possible that they could have play a role in explaining the results. Finally, as highlighted in the “Introduction,” the current study we evaluated BA while further studies are needed in order to assess if results can be generalized to other aspects of ToM.

## Conclusion

Three goals have been addressed in this study. The first was to evaluate the relation between syntax and ToM in adults using a dual task paradigm. Our aim was to understand if language is useful for ToM only during development or over the lifespan. Our results do not support the *Reasoning* account in the NT group which rather upholds the *Emergence* account, whereas in the AS group results were indirectly in favor of the *Reasoning* account. Our interpretation is that NT would need language during childhood only in order to develop their ToM abilities (Varley and Siegal, 2000; Varley et al., 2001; Apperly et al., 2006; Newton and de Villiers, 2007; Forgeot d’Arc and Ramus, 2011), while language in adults with AS could still be useful to ToM. Finally, syntax involving the embedding of a proposition with an independent truth-value (i.e., CS and *Misrepresentation* account) appears to be more important than other instances of syntactic embedding (i.e., RS and *Metarepresentation* account). This could suggest that adults with AS use verbally mediated strategies to compensate their ToM deficits.

## Ethics Statement

This study was carried out in accordance with the recommendations of the local ethics committee for non-interventional research, with written informed consent from all subjects. All subjects gave written informed consent in accordance with the Declaration of Helsinki. The protocol was approved by the CERNI Grenoble.

## Author Contributions

MCB, MP-B, SD, AR, and MB designed research; MCB performed research; MCB and MP-B analyzed data; MCB, MP-B, SD, AR, and MB wrote the paper.

## Conflict of Interest Statement

The authors declare that the research was conducted in the absence of any commercial or financial relationships that could be construed as a potential conflict of interest.

## Footnotes

1 We obtained a non-significant effect of Gender regarding RTs [*F*(1,70) < 1] and %ER [*F*(1,61) = 1.10, *p* = 0.30] whereas the main effect of Gender on d’ was significant [*F*(1,70) = 5.55, *p* = 0.02] with males obtaining higher d’ (Mean = 1.40, *SD* = 0.07) compared to women (Mean = 1.12, *SD* = 0.07). Nevertheless, the Interference × Gender interaction was non-significant [*F*(3,210) < 1] as the ±ToM × Gender interaction [*F*(1,70) = 2.70, *p* = 0.10] and the Interference × ToM × Gender [*F*(3,210) = 1.35, *p* = 0.26].
